# The role of artificial intelligence in maternal and child health: Progress, controversies, and future directions

**DOI:** 10.1371/journal.pdig.0000938

**Published:** 2025-07-17

**Authors:** Audêncio Victor

**Affiliations:** 1 Public Health Postgraduate Program, School of Public Health, University of São Paulo, São Paulo, Brazil; 2 Department of Nutrition, Ministry of Health of Mozambique, Zambezia, Mozambique; 3 Faculty of Epidemiology and Population Health, London School of Hygiene & Tropical Medicine, London, United Kingdom; Stanford University School of Medicine, UNITED STATES OF AMERICA

## Abstract

This debate paper examines the transformative potential of Artificial Intelligence (AI), specifically through Machine Learning (ML), in enhancing preventive measures in maternal and child health (MCH). With the proliferation of Big Data, ML has become crucial in handling complex, non-linear interactions among health determinants to not only predict but also prevent adverse outcomes. This paper underscores AI’s applications in early interventions that could decrease the incidence of MCH issues. It reviews technological advancements while addressing ethical, practical, and data-related challenges in applying AI in preventive healthcare. Emphasis is placed on recent supervised, unsupervised, and reinforcement learning applications that significantly advance preventive care, particularly in low-resource settings. The manuscript discusses the development of AI models for early diagnosis, comprehensive risk assessments, and customized preventive interventions, while highlighting challenges like data diversity, privacy issues, and integrating multimodal health data.

## Introduction

The integration of Artificial Intelligence (AI) into healthcare, especially in maternal and child health (MCH), marks a critical advancement with profound implications for preventive care and system efficiency. Machine Learning (ML), a subset of AI, leverages vast datasets to reveal complex relationships among health determinants that traditional methods cannot decipher [1,2]. The potential of ML in MCH has expanded with the advent of Big Data. These models can now process both structured and unstructured data, manage multifactorial interactions, and provide predictive insights crucial for early and preventive healthcare interventions [3]. Recent advances in ML have significantly enhanced predictive performance. This review systematically explores ML approaches to predicting preeclampsia, offering an overview of these enhancements. Notably, the models demonstrated high predictive performance with AUCs ranging from 0.860 to 0.973, utilizing predictors such as maternal characteristics, medical history, and prenatal care data. These findings underscore the potential for early intervention in maternal healthcare, leveraging ML to identify risks of preeclampsia from routine early pregnancy information [4].

MCH indicators are vital for assessing societal progress. Annually, preventable conditions cause approximately 2.9 million maternal and 5.2 million child deaths [5]. These deaths are preventable with access to quality healthcare [6,7]. Effective preventive strategies, enabled by AI, could dramatically improve access to quality care, especially in underserved areas [8]. However, as AI technology evolves, addressing issues of data diversity, model fairness, ethical usage, and patient privacy becomes imperative to avoid disparities and ensure equitable health benefits across different socioeconomic backgrounds.

### Machine learning

ML is known as a subfield of AI that aims to extract knowledge from large amounts of data, where algorithms are taught from previous examples [9,10]. This field has been growing in recent years due to the exponential increase in structured and unstructured data, also known as Big Data, which makes ML approaches increasingly important in data treatment and analysis, as traditional methods sometimes rely on conservative assumptions [11,12]. Additionally, in health research, it is important to understand the broad spectrum of specific phenotypes to make better predictions, which is possible through ML, allowing better phenotyping using multimodal data such as imaging, laboratory tests, clinical diagnosis, and genetics [13]. These models rely entirely on human intervention for pre-processing raw data, labeled or unlabeled, to estimate and compare known responses to determine accuracy. This is how the ML technique trains and builds models that help the machine replicate human behavior [9]. In MCH, ML is increasingly being utilized to predict complications such as pre-eclampsia, identify key variables for pregnancy outcomes, and analyze ultrasound images for fetal anomalies [9,14–16]. Multimodal data fusion, ML can produce comprehensive phenotyping and enhance predictions related to pregnancy complications, thereby supporting clinicians in early interventions.

ML techniques, categorized into supervised, unsupervised, semi-supervised, and reinforcement learning (RL), have distinct applications in MCH that cater to various predictive and preventive healthcare needs [17,18]. Supervised learning, this technique involves training algorithms on labeled datasets to predict outcomes. In the context of MCH, supervised learning algorithms have been successfully applied to predict high-risk pregnancies and adverse outcomes, such as premature births, pre-eclampsia, and gestational diabetes [10,19]. For example, using historical health data, these models can forecast the likelihood of complications, enabling early intervention strategies [20,21]. Meanwhile, unsupervised learning excels in identifying patterns and clusters within data without prior labeling. This method is particularly useful in segmenting patient populations into risk categories based on their health records, which aids in tailoring preventive measures and monitoring programs. In the MCH field, it has been applied to cluster patient data to identify high-risk groups or to process large-scale image data, such as MRIs, aiding in the detection of fetal anomalies [22,23]. In a groundbreaking study, convolutional neural networks were effectively utilized to interpret Cardiotocography signals, achieving high predictive accuracy for detecting fetal hypoxia, a critical condition that requires timely medical intervention. Additionally, techniques such as neural networks have been employed to enhance the accuracy of early detection models for Intrauterine Growth Restriction using ultrasound biometric measurements, demonstrating significant potential for AI in prenatal care [24]. RL is utilized to optimize decision-making in real-time by learning from the consequences of previous actions. In MCH, an RL model could dynamically adjust treatment plans for conditions like gestational diabetes by continuously learning from the patient’s responses to dietary adjustments, medication, and other interventions, thus maintaining optimal health conditions throughout pregnancy [25]. In MCH, an RL model could be applied to optimize treatment plans for gestational diabetes. This model would iteratively learn the most effective interventions, such as medication, dietary adjustments, and exercise recommendations by analyzing real-time feedback on the mother’s glucose levels, fetal health indicators, and other health outcomes. Each successful outcome, such as maintaining target blood glucose levels or preventing complications, reinforces the system, allowing the agent to adjust and refine future recommendations [26]. Over time, the model becomes increasingly accurate in providing personalized treatment plans that support the health of both mother and child. Despite these advances, the limited access to high-quality, diverse data, especially in low-resource settings, poses challenges. Approaches like transfer learning, which adapts models trained on well-resourced datasets for use in low-resource environments, and federated learning, which enables collaborative model training across institutions while preserving data privacy [27], are critical for expanding ML’s applicability globally.

### IA for maternal-infant health outcome prediction

AI-driven models are revolutionizing MCH care by enabling early and precise predictions of various health conditions, addressing key priorities in public health. These models leverage data from diverse sources, including maternal demographics, laboratory results, and prenatal imaging, to forecast complications such as pre-eclampsia, gestational diabetes, and neonatal disorders. Early detection facilitated by AI not only allows for timely medical interventions but also improves clinical decision-making, significantly reducing adverse health outcomes for mothers and infant [1]. Although perinatal health indicators such as maternal and neonatal mortality, low birth weight, and preterm birth have shown improvements globally, disparities remain stark, particularly in low-resource settings where access to advanced healthcare is limited [20,28].

In neonatal care, AI models predict conditions like sepsis and jaundice by analyzing physiological data from the first hours after birth, enabling proactive management of critical cases [9,10]. Similarly, ML models for gestational diabetes assess historical health records and current pregnancy data to identify risks early, guiding preventive strategies such as lifestyle interventions [29]. These predictive tools extend to evaluating birth weight, infant mortality, and complications in in vitro fertilization, utilizing comprehensive analyses of maternal health and environmental influences [30–32]. Additional applications include predicting emergency cesarean sections [33], and enhancing the prediction of perinatal mortality in vulnerable populations [34].

However, a critical gap persists in the application of AI to low- and middle-income countries (LMICs), where MCH challenges are often compounded by limited infrastructure. The majority of existing studies are concentrated in high-income countries, limiting the generalizability of predictive models to diverse populations [9]. Addressing this gap demands the adaptation of AI algorithms to LMIC contexts through data augmentation, integration of locally relevant factors, and partnerships with international health organizations. Such efforts would enhance dataset diversity and ensure models are culturally and socioeconomically relevant, ultimately fostering equitable healthcare and reducing global health disparities.

Using data from the National Institute of Child Health and Human Development (NICHD) Neonatal Research Network’s Neonatal Research Database [35], demonstrated that prenatal or pre-delivery variables exhibited lower predictive accuracy for neonatal mortality compared to immediate post-delivery variables. The study emphasized the critical role of birth weight in predicting neonatal outcomes, highlighting it as a dominant factor, while other variables were found to have relatively limited predictive value. In practical applications, real-time fetal electrocardiogram recordings allow for continuous monitoring of fetal status, empowering both patients and physicians. Similarly, AI-based sensors for monitoring blood glucose and blood pressure have proven particularly beneficial in low-resource settings, addressing gaps in MCH. These tools gained heightened significance during the COVID-19 pandemic, when healthcare systems were overwhelmed, and patients with pre-existing conditions faced challenges in accessing timely care and follow-ups [36]. One of the most promising AI developments in this context is mobile health (mHealth), which expands access to care through portable and scalable solutions.

MCH increasingly relies on diverse algorithms tailored to specific tasks, such as image analysis, risk prediction, and clinical decision-making. Supervised learning dominates, leveraging classification and regression models for predictive applications. Meanwhile, unsupervised learning methods, like clustering and dimensionality reduction, enable pattern recognition in complex datasets, including imaging [36]. Emerging methodologies are further expanding AI’s potential in healthcare. RL, for example, applies a trial-and-error approach to optimize personalized interventions based on patient-specific responses [25,26]. Ensemble learning and multimodal data fusion techniques are gaining traction, integrating diverse datasets such as electronic health records, imaging, and genomic information to improve predictive accuracy in MCH [9,37]. Recent studies underscore the effectiveness of boosting algorithms for handling tabular data, demonstrating superior performance across various tasks [38,39]. The latest innovation, TabPFN, has further solidified the role of boosting in advancing predictive capabilities in MCH [37].

Trust remains a cornerstone of AI integration into healthcare. As such, explainable AI (XAI) has emerged as a critical focus, ensuring transparency in decision-making processes. This transparency is vital for healthcare providers and patients to understand and rely on AI-driven recommendations. Techniques like decision trees and interpretable neural network frameworks offer clarity on algorithmic predictions, making them particularly valuable in sensitive fields like MCH [40,41]. By balancing technological sophistication with interpretability, AI systems can better address the nuanced needs of MCH, fostering equity and trust in healthcare delivery.

## The future of AI in MCH

### Importance of MCH algorithms in remote regions

The use of algorithms in MCH in remote regions can play a crucial role in improving care and reducing health disparities. Algorithms can be applied in various areas, from early diagnosis of complications to continuous monitoring of the health of pregnant women and newborns. In remote regions where access to healthcare services is limited, algorithms can help identify risk factors and potential complications during prenatal care [42]. For example, algorithms can be used to analyze demographic data, medical history, and test results to identify pregnant women who are more likely to develop complications such as preeclampsia or gestational diabetes [43]. This allows healthcare professionals to prioritize care and provide appropriate interventions more efficiently. Additionally, algorithms can be used to continuously monitor the health of pregnant women and newborns. Mobile devices and sensors can collect data such as blood pressure, fetal heart rate, and baby movements, and transmit them to healthcare professionals [3]. Algorithms can analyze this data in real-time and alert professionals to any anomalies or early warning signs. This enables quick and timely intervention, reducing the risk of serious complications. The use of algorithms in MCH can also facilitate patient self-management [20]. For example, mobile applications can provide personalized information on prenatal care, proper nutrition, and recommended physical exercises.

Furthermore, limited access to health data in these regions can be a significant obstacle to the effective implementation of ML algorithms. This is where techniques like transfer learning and federated learning play a crucial role. Transfer learning allows pre-trained ML models on large datasets to be adapted for specific tasks in MCH, saving time and resources [44]. Federated learning is an innovative technique that enables training models on distributed data across multiple locations while preserving patient data privacy [27], by employing these advanced techniques, it is possible to significantly improve the efficiency and accuracy of diagnoses and treatments, contributing to the improvement of MCH care in geographically isolated areas. For instance, a project in rural India utilized mobile health units equipped with AI-driven diagnostic tools that used federated learning to improve prenatal care. The AI system helped in early detection of gestational diabetes by analyzing patient data collected on-site, which was then processed collectively without ever leaving the device, ensuring data privacy and enabling timely interventions [45].

### Controversies and challenges

Despite the transformative potential of AI, several controversies and challenges persist, particularly concerning data biases, ethical use, and equitable access. A primary concern is the lack of data diversity, which can result in models trained on non-representative datasets yielding biased predictions. These biases may disproportionately affect underrepresented populations [9,10,46,47]. Addressing this issue is crucial to ensuring equitable health benefits across all socioeconomic backgrounds. This is underscored by ongoing efforts to adhere to ethical guidelines such as SPIRIT-AI and CONSORT-AI [48]. Ethical deployment of AI in healthcare necessitates stringent data governance frameworks to manage patient consent and privacy concerns effectively, ensuring that AI tools are used responsibly and transparently.

Collaborative endeavors with local health organizations and community-based data collection are strategies that can help mitigate these biases, ensuring that AI applications in MCH are inclusive and fair. Moreover, the reliance on automated decision-making systems in MCH raises significant ethical implications, particularly in high-stakes scenarios where AI-generated recommendations may carry life-altering consequences. It is critical to retain human oversight to validate these AI decisions. Implementing mechanisms for accountability and transparency, including regular audits of AI systems, is essential to maintain trust and ensure the accuracy of these technologies.

### Implications for public health policy and practice

The integration of AI into MCH has profound implications for public health policy and practice, shaping not only clinical outcomes but also the broader healthcare landscape. To maximize AI’s transformative potential, it is imperative to establish policies that promote the sustainable development, equitable distribution, and ethical use of AI technologies. Governments and health organizations must prioritize investments in infrastructure, such as mobile networks and affordable health-monitoring devices, to enable the adoption of AI solutions in remote and underserved areas. Collaborations with international health organizations like the WHO are vital for ensuring the inclusivity and efficacy of AI in MCH. These partnerships can help diversify datasets, ensuring that AI models are trained on data representing various populations, thereby mitigating biases and enhancing global applicability. For example, integrating data from low-resource settings can refine models to better address health disparities. Concurrently, capacity-building programs for healthcare providers in resource-limited regions are essential. Such programs should focus on equipping local healthcare workers with the knowledge and skills to effectively deploy and utilize AI tools, thereby improving care quality and reducing inequalities in healthcare delivery.

AI, particularly ML, holds immense potential to revolutionize MCH by enhancing predictive capabilities, enabling early interventions, and providing robust decision support for healthcare providers. Advancements in data integration and predictive analytics allow AI to address critical challenges, such as reducing preventable maternal and infant mortality and morbidity rates. Real-world applications, such as AI-based screening for gestational diabetes or predicting neonatal sepsis, demonstrate its utility in improving health outcomes. However, realizing this potential requires addressing significant challenges, including limited access to diverse and high-quality datasets, ethical concerns regarding patient privacy, and infrastructural barriers in low-resource settings.

Future research should prioritize refining AI methodologies to ensure transparency, accuracy, and applicability across diverse contexts. Explainable AI and multimodal data fusion techniques are particularly promising for enhancing the interpretability and reliability of AI-driven insights. Moreover, policymakers and healthcare institutions must establish frameworks that promote the ethical and responsible use of AI. These frameworks should emphasize data privacy, equitable access, and the development of culturally sensitive AI tools. Efforts to bridge data and resource gaps, facilitated through international collaboration, are critical to achieving these objectives.

In conclusion, leveraging AI’s full potential in MCH will require ongoing innovation, strategic investment, and cross-sector collaboration. By fostering partnerships between researchers, healthcare providers, policymakers, and communities, we can drive forward AI solutions that not only improve health outcomes but also contribute to a more inclusive and sustainable global health ecosystem. Through these concerted efforts, AI can become a cornerstone of equitable healthcare delivery, particularly for vulnerable populations worldwide.

## References

[1] Islam MN, Mustafina SN, Mahmud T, Khan NI. Machine learning to predict pregnancy outcomes: a systematic review, synthesizing framework and future research agenda. BMC Pregnancy Childbirth. 2022;22(1):348. doi: 10.1186/s12884-022-04594-2 35546393 PMC9097057

[2] Lee K-S, Ahn KH. Application of artificial intelligence in early diagnosis of spontaneous preterm labor and birth. Diagnostics (Basel). 2020;10(9):733. doi: 10.3390/diagnostics10090733 32971981 PMC7555184

[3] Shukla VV, Eggleston B, Ambalavanan N, McClure EM, Mwenechanya M, Chomba E, et al. Predictive modeling for perinatal mortality in resource-limited settings. JAMA Netw Open. 2020;3(11):e2026750. doi: 10.1001/jamanetworkopen.2020.26750 33206194 PMC7675108

[4] Ranjbar A, Montazeri F, Ghamsari SR, Mehrnoush V, Roozbeh N, Darsareh F. Machine learning models for predicting preeclampsia: a systematic review. BMC Pregnancy Childbirth. 2024;24(1):6. doi: 10.1186/s12884-023-06220-1 38166801 PMC10759509

[5] WHO. Fact sheet on child health. Genebra; 2022. Available from: https://www.who.int/news-room/fact-sheets/detail/child-health

[6] Mannava P, Durrant K, Fisher J, Chersich M, Luchters S. Attitudes and behaviours of maternal health care providers in interactions with clients: a systematic review. Global Health. 2015;11:36. doi: 10.1186/s12992-015-0117-9 26276053 PMC4537564

[7] Brockerhoff M, Hewett P. Inequality of child mortality among ethnic groups in sub-Saharan Africa. Bull World Health Organ. 2000;78(1):30–41. 10686731 PMC2560588

[8] Erku D, Khatri R, Endalamaw A, Wolka E, Nigatu F, Zewdie A, et al. Digital health interventions to improve access to and quality of primary health care services: a scoping review. Int J Environ Res Public Health. 2023;20(19):6854. doi: 10.3390/ijerph20196854 37835125 PMC10572344

[9] Islam MN, Mustafina SN, Mahmud T, Khan NI. Machine learning to predict pregnancy outcomes: a systematic review, synthesizing framework and future research agenda. BMC Pregnancy Childbirth. 2022;22(1):348. doi: 10.1186/s12884-022-04594-2 35546393 PMC9097057

[10] Lee K-S, Ahn KH. Application of artificial intelligence in early diagnosis of spontaneous preterm labor and birth. Diagnostics (Basel). 2020;10(9):733. doi: 10.3390/diagnostics10090733 32971981 PMC7555184

[11] Jordan MI, Mitchell TM. Machine learning: trends, perspectives, and prospects. Science. 2015;349(6245):255–60. doi: 10.1126/science.aaa8415 26185243

[12] Loring Z, Mehrotra S, Piccini JP. Machine learning in “big data”: handle with care. Europace. 2019;21(9):1284–5. doi: 10.1093/europace/euz130 31106325 PMC6736339

[13] Davidson L, Boland MR. Towards deep phenotyping pregnancy: a systematic review on artificial intelligence and machine learning methods to improve pregnancy outcomes. Brief Bioinform. 2021;22(5):bbaa369. doi: 10.1093/bib/bbaa369 33406530 PMC8424395

[14] Zou Q, Qu K, Luo Y, Yin D, Ju Y, Tang H. Predicting Diabetes mellitus with machine learning techniques. Front Genet. 2018;9:515. doi: 10.3389/fgene.2018.00515 30459809 PMC6232260

[15] Islam MN, Mahmud T, Khan NI, Mustafina SN, Islam AKMN. Exploring machine learning algorithms to find the best features for predicting modes of childbirth. IEEE Access. 2021;9:1680–92. doi: 10.1109/access.2020.3045469

[16] Aishwarja AI, Eva NJ, Mushtary S, Tasnim Z, Khan NI, Islam MN. Exploring the machine learning algorithms to find the best features for predicting the breast cancer and its recurrence. 2021.

[17] Saini A, Meitei AJ, Singh J. Machine learning in healthcare: a review. SSRN J. 2021. doi: 10.2139/ssrn.3834096

[18] Ayodele TO. Machine learning overview. New Adv Mach Learn. 2010;2:9–18.

[19] Włodarczyk T, Płotka S, Szczepański T, Rokita P, Sochacki-Wójcicka N, Wójcicki J, et al. Machine learning methods for preterm birth prediction: a review. Electronics. 2021;10(5):586. doi: 10.3390/electronics10050586

[20] Batista AFM, Diniz CSG, Bonilha EA, Kawachi I, Chiavegatto Filho ADP. Neonatal mortality prediction with routinely collected data: a machine learning approach. BMC Pediatr. 2021;21(1):322. doi: 10.1186/s12887-021-02788-9 34289819 PMC8293479

[21] Pereira S, Portela F, Santos MF, Machado J, Abelha A. Predicting type of delivery by identification of obstetric risk factors through data mining. Proc Comput Sci. 2015;64:601–9. doi: 10.1016/j.procs.2015.08.573

[22] Xu D, Tian Y. A comprehensive survey of clustering algorithms. Ann Data Sci. 2015;2(2):165–93. doi: 10.1007/s40745-015-0040-1

[23] Alam MS, Rahman MM, Hossain MA, Islam MK, Ahmed KM, Ahmed KT, et al. Automatic Human brain tumor detection in MRI image using template-based K means and improved fuzzy C means clustering algorithm. BDCC. 2019;3(2):27. doi: 10.3390/bdcc3020027

[24] Mennickent D, Rodríguez A, Opazo MC, Riedel CA, Castro E, Eriz-Salinas A, et al. Machine learning applied in maternal and fetal health: a narrative review focused on pregnancy diseases and complications. Front Endocrinol (Lausanne). 2023;14:1130139. doi: 10.3389/fendo.2023.1130139 37274341 PMC10235786

[25] Ding Z, Huang Y, Yuan H, Dong H. Introduction to reinforcement learning. 2020.

[26] Yu C, Liu J, Nemati S, Yin G. Reinforcement learning in healthcare: a survey. ACM Comput Surv. 2021;55(1):1–36. doi: 10.1145/3477600

[27] Yang Q, Liu Y, Chen T, Tong Y. Federated machine learning: concept and applications. 2019. Available from: http://arxiv.org/abs/1902.04885

[28] Ramakrishnan R, Rao S, He J-R. Perinatal health predictors using artificial intelligence: a review. Womens Health (Lond). 2021;17. doi: 10.1177/17455065211046132 34519596 PMC8445524

[29] Lakshmi BN, Indumathi TS, Ravi N. A study on C.5 decision tree classification algorithm for risk predictions during pregnancy. Proc Technol. 2016;24:1542–9. doi: 10.1016/j.protcy.2016.05.128

[30] Tesfaye B, Atique S, Elias N, Dibaba L, Shabbir S-A, Kebede M. Determinants and development of a web-based child mortality prediction model in resource-limited settings: a data mining approach. Comput Methods Programs Biomed. 2017;140:45–51. doi: 10.1016/j.cmpb.2016.11.013 28254089

[31] Ghaderighahfarokhi S, Sadeghifar J, Mozafari M. A model to predict low birth weight infants and affecting factors using data mining techniques. JBRMS. 2018;5(3):1–8. doi: 10.29252/jbrms.5.3.1

[32] Hassan MR, Al-Insaif S, Hossain MI, Kamruzzaman J. A machine learning approach for prediction of pregnancy outcome following IVF treatment. Neural Comput Appl. 2018;32(7):2283–97. doi: 10.1007/s00521-018-3693-9

[33] Guan P, Tang F, Sun G, Ren W. Prediction of emergency cesarean section by measurable maternal and fetal characteristics. J Investig Med. 2020;68(3):799–806. doi: 10.1136/jim-2019-001175 31980540 PMC7057850

[34] Mboya IB, Mahande MJ, Mohammed M, Obure J, Mwambi HG. Prediction of perinatal death using machine learning models: a birth registry-based cohort study in northern Tanzania. BMJ Open. 2020;10(10):e040132. doi: 10.1136/bmjopen-2020-040132 33077570 PMC7574940

[35] Shukla VV, Eggleston B, Ambalavanan N, McClure EM, Mwenechanya M, Chomba E, et al. Predictive modeling for perinatal mortality in resource-limited settings. JAMA Netw Open. 2020;3(11):e2026750. doi: 10.1001/jamanetworkopen.2020.26750 33206194 PMC7675108

[36] Oprescu AM, Miro-Amarante G, Garcia-Diaz L, Beltran LM, Rey VE, Romero-Ternero Mc. Artificial intelligence in pregnancy: a scoping review. IEEE Access. 2020;8:181450–84. doi: 10.1109/access.2020.3028333

[37] McElfresh D, Khandagale S, Valverde J, Ramakrishnan G, Goldblum M, White C. When do neural nets outperform boosted trees on tabular data? arXiv preprint arXiv:230502997. 2023.

[38] Shwartz-Ziv R, Armon A. Tabular data: deep learning is not all you need. Information Fusion. 2022;81:84–90. doi: 10.1016/j.inffus.2021.11.011

[39] Borisov V, Leemann T, Sebler K, Haug J, Pawelczyk M, Kasneci G. Deep neural networks and tabular data: a survey. IEEE Trans Neural Netw Learn Syst. 2024;35(6):7499–519. doi: 10.1109/TNNLS.2022.3229161 37015381

[40] Rodríguez-Pérez R, Bajorath J. Interpretation of machine learning models using shapley values: application to compound potency and multi-target activity predictions. J Comput Aided Mol Des. 2020;34(10):1013–26. doi: 10.1007/s10822-020-00314-0 32361862 PMC7449951

[41] Patel SS. Explainable machine learning models to analyse maternal health. Data Knowl Eng. 2023;146:102198. doi: 10.1016/j.datak.2023.102198

[42] Mboya IB, Mahande MJ, Mohammed M, Obure J, Mwambi HG. Prediction of perinatal death using machine learning models: a birth registry-based cohort study in northern Tanzania. BMJ Open. 2020;10(10):e040132. doi: 10.1136/bmjopen-2020-040132 33077570 PMC7574940

[43] Guedalia J, Lipschuetz M, Novoselsky-Persky M, Cohen SM, Rottenstreich A, Levin G, et al. Real-time data analysis using a machine learning model significantly improves prediction of successful vaginal deliveries. Am J Obstet Gynecol. 2020;223(3):437.e1-437.e15. doi: 10.1016/j.ajog.2020.05.025 32434000

[44] Zhuang F, Qi Z, Duan K, Xi D, Zhu Y, Zhu H, et al. A comprehensive survey on transfer learning. Proc IEEE. 2021;109(1):43–76. doi: 10.1109/jproc.2020.3004555

[45] Oprescu AM, Miro-Amarante G, Garcia-Diaz L, Beltran LM, Rey VE, Romero-Ternero Mc. Artificial intelligence in pregnancy: a scoping review. IEEE Access. 2020;8:181450–84. doi: 10.1109/access.2020.3028333

[46] Ramakrishnan R, Rao S, He J-R. Perinatal health predictors using artificial intelligence: a review. Womens Health (Lond). 2021;17. doi: 10.1177/17455065211046132 34519596 PMC8445524

[47] Grote T, Keeling G. Enabling fairness in healthcare through machine learning. Ethics Inf Technol. 2022;24(3):39. doi: 10.1007/s10676-022-09658-7 36060496 PMC9428374

[48] Muralidharan V, Schamroth J, Youssef A, Celi LA, Daneshjou R. Applied artificial intelligence for global child health: addressing biases and barriers. PLOS Digit Health. 2024;3(8):e0000583. doi: 10.1371/journal.pdig.0000583 39172772 PMC11340888

